# Analysis of scientific and technological trends in the incorporation of activated carbon in advanced oxidation processes—a bibliometric study

**DOI:** 10.1007/s11356-023-31120-4

**Published:** 2023-12-23

**Authors:** Diego Montenegro-Apraez, Fiderman Machuca-Martínez

**Affiliations:** Escuela de Ingeniería Química, Universidad del Valle, Calle 13 No 100-00, AA, 25360 Cali, Colombia

**Keywords:** Advanced oxidation processes, Activated carbon, Adsorption, Catalyst, Catalyst support, Electrode

## Abstract

There is high interest in the development of water pollution remediation technologies. Advanced oxidation processes (AOPs) are a promising alternative for the degradation of organic compounds; however, these technologies have been limited mainly by high operating costs and, in some cases, by forming byproducts, which can be more hazardous than the original pollutants. Activated carbon (AC) is a porous material that can be combined with AOP systems in various ways, given its adsorbent and catalytic characteristics. In addition, AC is a flexible, adaptable, and low-cost material. This article presents a bibliometric analysis of AOPs incorporating CA in scientific research and patents; the Scopus database was used to obtain patents and Orbit Express for patents. The most investigated AOPs incorporating AC are photocatalysis processes, Fenton processes, persulfate-based AOP, electrochemical processes, and ozonation. However, it is the persulfate-based AOP that has seen the greatest growth in scientific publications in recent years; this great interest can be related to the synergy that the process has with AC, allowing the degradation of contaminants via radical and non-radical. According to the maturity analysis of scientific publications, photocatalysis, Fenton, electrochemistry, ozonation, and persulfate technologies are in a growth stage and will reach maturity in 2034, 2042, 2040, 2034, and 2035, respectively; these technologies coupled with AC are expected to generate a greater number of patents when they reach maturity.

## Introduction

Advanced oxidation processes (AOPs) are chemical treatments that can be divided into homogeneous and heterogeneous processes. Homogeneous processes proceed in a single phase, where hydroxyl radicals (^•^OH) are generated from ozone, hydrogen peroxide, or other oxidants; homogeneous processes include electro-Fenton, ultrasound/O_3_, ultrasound/H_2_O_2_, oxidation by humid air, electrochemical oxidation, UV photolysis, UV/H_2_O_2_, photo-Fenton, and UV/O_3_/H_2_O_2_. Heterogeneous processes are characterized by the use of catalysts and include catalytic ozonation, H_2_O_2_/catalyst, persulfate/catalyst, photoelectrocatalysis, UV/O_3_/TiO_2_, UV/H_2_O_2_/TiO_2_, UV/H_2_O_2_/catalyst, and UV/persulfate/catalyst processes (Taoufik et al. 2021).

**Fig. 1:**
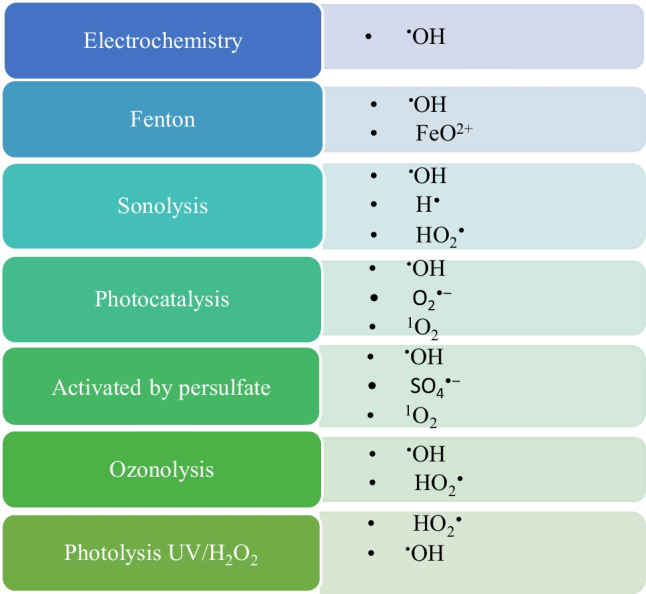
Some AOPs and the reactive oxygen species involved

AOPs generally use hydroxyl radicals (^•^OH) as oxidants, which have excellent performance for the degradation of different organic pollutants (Zhang et al. 2022). In addition to ^•^OH, other reactive oxygen species, such as SO_4_^•–^, O_2_^*−^, ^1^O_2_, and H_2_O^*^, can degrade pollutants (Rayaroth et al. 2022). The AOPs and the reactive oxygen species involved are described in Fig. 1.

Some AOPs, such as ozonation, photodegradation, and electro-Fenton, have shown excellent efficiency in the degradation of different organic compounds; however, they have been hampered by the high cost of energy, high operating costs, and sometimes the formation of byproducts that may be more toxic than the original pollutants (Taoufik et al. 2021). This has led to evaluating AOPs systems combined with other processes, considering environmental friendliness, feasibility, and cost-effectiveness. For example, hybrid methods based on cavitation with AOPs have demonstrated higher oxidation capacity; studies have shown their potential for industrial-scale use for water and wastewater treatment (Fedorov et al. 2022). Badmus et al. (2018) mention the advantages of coupled systems, which are generally energy conserving, are efficient, and improve the limitations that a process alone may have; the above allows them to be commercially applicable; in this study, the authors mention the synergistic effect between AOPs systems with hydrodynamic cavitation. Tang and Mao (2023) reviewed the physicochemical and biological treatment technologies available for removing 1,4-dioxane from water; the authors propose that coupled processes have great potential for synergistic removal of the pollutant at lower costs. Fedorov et al. (2023) evaluated the degradation of 1,4-dioxane using hydrodynamic cavitation to activate sodium percarbonate and ozone; the authors obtained a degradation greater than 99%. Phan et al. (2023) evaluated the combination of AOPs coupled with activated carbon (AC) to remove natural organic matter, achieving a reduction of DOC by 40% and UV254 by 52%; these results were obtained using the O_3_-UV-AC system in real waters. Gloria et al. (2023) also used AC as a support for TiO2, which allowed improving the photodegradation of paracetamol, achieving 95% degradation of the pollutant.

One of the technologies that have been highly studied and that can be coupled in different ways to AOPs is activated carbon (AC). AC is a material that contains a well-developed internal pore structure. The large surface area, high porosity (micro-, meso-, and macropores), and wide spectrum of functional groups present on the surface make it a versatile material, which is why it is applied as an adsorbent in different fields (Bhatnagar et al. 2013). Water treatment stands out among these fields, where AC has been found to be efficient for the adsorption of organic pollutants, heavy metals, and other inorganic pollutants. It is also evidenced that AC is highly efficient in the adsorption of emerging contaminants. Rodriguez-Narvaez et al. (2017) report removal efficiencies higher than 80% of different emerging contaminants, which depend on the characteristics of the AC and the operating conditions (Rodriguez-Narvaez et al. 2017).

The excellent adsorbent performance of AC is not the only quality of this material considered when it is applied in AOP systems. AC also has high performance as a catalyst and catalyst support, showing stability in acidic and basic media, a low corrosion capacity, hydrophobic behavior, and good recovery of the reaction mixture (Khan 2021). The characteristics of AC are determined by the preparation method (activation and carbonization process) and raw material, which influence the porous structure and surface chemistry of AC (Rodríguez 1998). Moreover, modification treatments can be used to generate different characteristics of AC and thus adjust the physicochemical properties of the material. For example, functional groups can be added to the surface of ACs to improve their catalytic performance (Matos et al. 2017).

Thus, this article presents an overview of the work that has been developed on AOPs incorporating AC, both in scientific articles and patents. In addition, the collaborative relationship around scientific research between the countries and institutions involved is presented. Research trends in AOPs (photocatalysis, Fenton processes, electrochemical processes, ozonation, and persulfate-based AOPs) that incorporate AC as an adsorbent, catalyst, catalyst support, and electrode are also reviewed. Finally, the article includes an analysis of the technological maturity of the AOPs-AC investigated.

## Methodology

The data were obtained from scientific articles and patents through a search carried out on November 22, 2022. The Scopus database was used to obtain scientific articles, and Orbit Express was used for patents. The search terms used are detailed in Table 1. ORBIT ITELLIXIR software was used for data visualization and analysis.

**Table 1:** Search terms

T1	Articles	TITLE-ABS-KEY (((“advanced oxidation processes” OR “advanced oxidation process”) AND (“activated carbo*” OR “activated charcoal”)) AND NOT comparative) AND (LIMIT-TO (DOCTYPE, “ar”))
T2	Patents	(advanced oxidation processes OR advanced oxidation process) AND (activated carbon OR activated charcoal)

## Results and discussion

### Scientific research and patent production

**Fig. 2:**
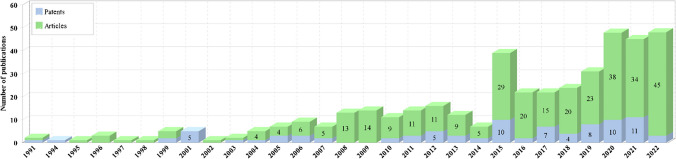
Evolution of the number of articles and patents

A total of 329 scientific articles and 89 patents were obtained in the search. Figure 2 shows the evolution of the number of articles and patents related to the incorporation of AC into AOPs. The publication of both articles and patents began in 1991. This could be explained by the fact that AOPs began to receive greater interest from the scientific community at the beginning of the 1990s. In 2004, interest in scientific publications began to increase slightly, although this growth is unstable.

**Fig. 3:**
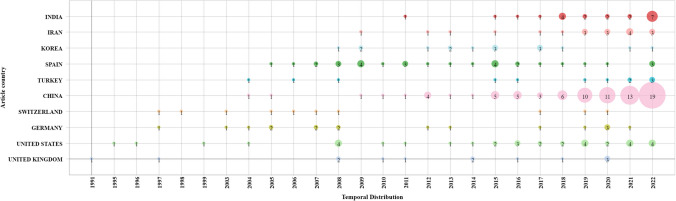
Distribution of scientific articles by country

**Fig. 4:**
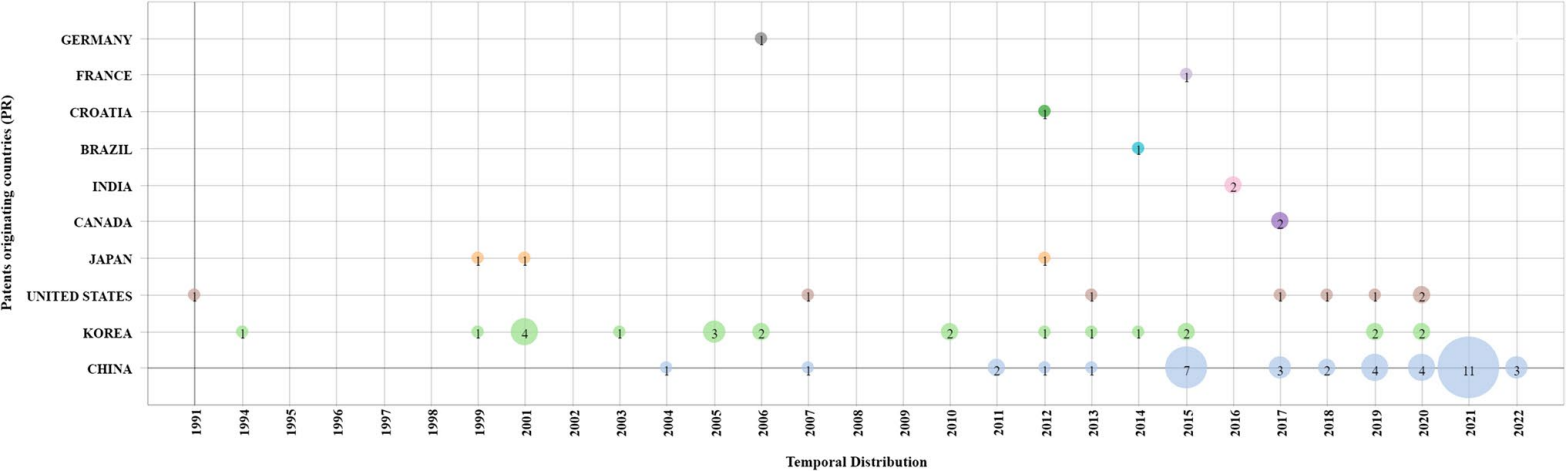
Distribution of patents by country

For scientific publications and patents, there is no highly predominant country regarding work on AOPs with AC. However, China is the country that stands out the most (83 publications of articles and 40 patents), which began to produce scientific articles and patents in 2004 (see Figs. 3 and 4). Although the consistent production of scientific articles began in 2009, China’s interest in scientific research and technological developments in this area could be influenced by government policies targeting climate change, such as laws focused on the prevention and control of water pollution, marine environmental protection, and water that have been enacted in the last two decades (Khan and Chang 2018).

The other countries that also stand out in the publication of scientific articles are the USA and Spain, which represent 10.6% and 9.4% of the total articles, respectively.

Before the emergence of China as a publisher of patents (between 1991 and 2010), Korea was the country that had published the largest number of patents; however, in this same period, Korea reported only 3 scientific articles but 16 patents (see Figs. 3 and 4). This situation could be interpreted as Korea focusing on technological development and not on basic or applied research in this field. It was only in 2008 that Korea began to generate scientific articles, but the rate of production was not consistent.

The countries with the highest number of patents are China with 44.9%, Korea with 25.8%, and the USA with 9%.

### Collaborative relationships between countries

**Fig. 5:**
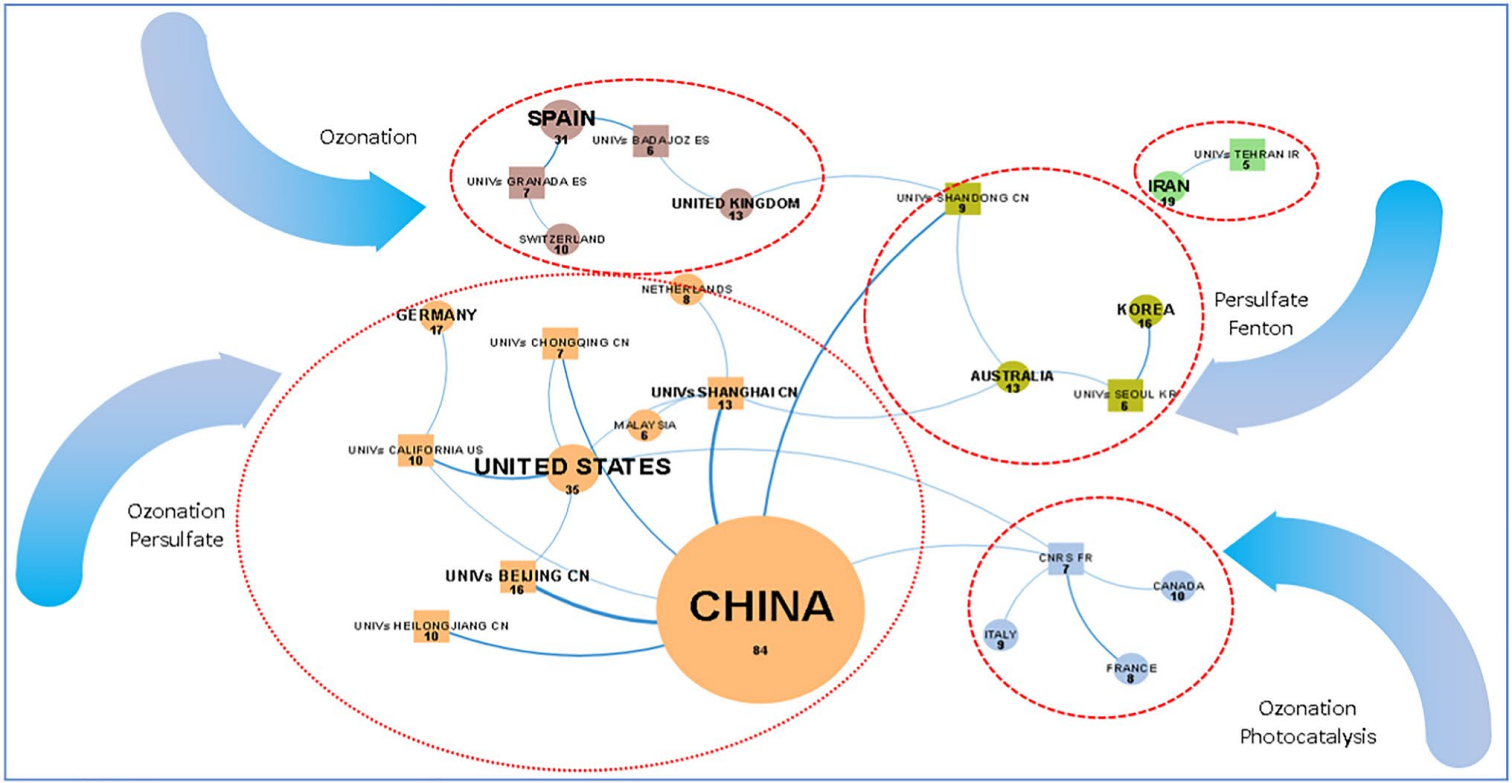
Network of collaboration between countries

Concerning the publication of scientific articles, Fig. 5 shows the collaboration networks between countries and the institutions that connect them. The largest cluster consists of China, the USA, Germany, the Netherlands, and Malaysia. Chinese universities are the largest collaborators in joint research. China connects with the USA through the University of Shanghai, the University of Beijing, the University of Chongquing, and the University of California. Through the University of Shanghai, China also connects with the Netherlands and Malaysia; this cluster focuses on ozonation and persulfate research.

Although it is one of the countries that publishes the most, Spain is only connected with Switzerland through the University of Granada and the UK through the University of Extremadura-Badajoz; this cluster focuses on ozonation research.

Australia is connected to China through the University of Shandong and Korea through Seoul University. This cluster focuses on research on persulfate and Fenton.

France is connected to China, Italy, Canada, and the USA through the National Center for Scientific Research (CNRS); this cluster focuses on research in ozonation and photocatalysis.

The AOPs with AC generating the most interest in collaborative research between countries have been ozonation and persulfate-based AOPs. In ozonation process research incorporating AC, the universities that lead these connections are the University of Beijing, the University of Extremadura-Badajoz, the University of Heilongjiang, the University of California, the National Center for Scientific Research, and the University of Granada. In research on persulfate-based AOPs incorporating AC, the universities leading these connections are the University of Beijing and the University of Shandong.

Table 2 shows the main authors’ affiliations according to published articles. It can be seen that universities in China have a strong connection with each other to conduct joint research, allowing China to become stronger in this area.

**Table 2:** Main authors

Country	University	Authors
China	University of Beijing	Li J
		Li S
		Li Xiuping
		Li Yuping
		Wang Jingyu
		Wang Xiaonan
		Wang Yujue
		Zhang Xiaoyuan
		Zhang Y
	University of Heilongjiang	Li J
		Lu J
		Ma J
		Wan J
		Wang L
		Wang S
		Wang Xiaonan
	University of Shandong	Chen Yuhui
		Liu Junjie
		Lu J
		Wang Xiaonan
		Wang Yujue
		Zhang Xiaoyuan
	University of Shanghai	Chen Yuhui
		Liu Junjie
		Liu Z
		Wang L
		Wang Xiaonan
		Wang Yujue
		Zhang Y
	University of Chongquing	Li Xiuping
		Li Yuping
		Liu Z
		Zhang Xiaoyuan
Spain	University of Granada	Méndez-díaz Jd
		Rivera-urtrilla J
		Sanchez-polo M
		Von gunten U
USA	University of California	Li Yuping
France	National Center for Scientific Research	Lu J
		Wang L

### Trends in research in AOPs

In the documents found in the search for scientific research and patents, the AOPs stand out: photocatalysis, Fenton, electrochemistry, ozonation, and persulfate, which incorporate AC as adsorbent, catalyst, catalyst support, and electrode.

#### Photocatalysis

Photocatalysis is based on accelerating the remediation process to degrade contaminants by photocatalytic materials by using a light or radiation source. Photocatalytic oxidation leads to the formation of hydroxyl radicals, which are strong oxidizing agents that can decompose several organic compounds (Saravanan et al. 2022). The studies on photocatalysis that have been widely carried out in recent decades are based on water treatment, CO_2_ reduction, nitrogen fixation, degradation of pollutants, and organic synthesis (Wu et al. 2022).

In the current search, 24 documents related to photocatalysis were found. AC applications in photocatalysis have registered scientific publications (79.2%) and patents (20.8%) since 2004, with 2022 being the year with the highest number of documents (three articles and two patents).

Within these 24 documents, it was found that there are publications that relate photocatalysis to other AOPs: photocatalysis – Fenton = 8 documents, photocatalysis – ozonation = 8 documents, photocatalysis – electrochemical processes = 3 documents, and photocatalysis – persulfate = 1 document. This demonstrates that most photocatalytic processes incorporating AC are not studied in isolation, but coupled to other AOPs.

According to the scientific articles found, the most prominent applications of AC in photocatalysis processes involve its use as a catalyst support, and it is also used as an adsorbent in processes before or after photocatalysis. However, there is no trend toward the evaluation of a particular pollutant (see Fig. 7).

Table 3 shows the articles published in 2022, where the applications of AC in the photocatalysis process (catalyst support and adsorbent) are identified. The three studies published this year use TiO_2_ as a catalyst; this is because TiO_2_ is the most widely researched photocatalyst due to its chemical stability, low cost, and eco-friendliness; however, this catalyst (TiO_2_) has its limitations since it is only sensitive to the UV range. AC as a support for TiO_2_ can help the photocatalytic activity by improving visible light absorption and charge separation. Furthermore, when TiO_2_ particles are supported on AC, these particles are well dispersed, resulting in high photocatalytic activity. On the other hand, the photogenerated electrons are trapped in the AC and pass to the molecules of interest, improving the photocatalytic capacity. Likewise, the presence of AC facilitates the adsorption of contaminants, which allow them to be close to the radicals generated to undergo photodegradation (Thambiliyagodage 2022). The efficiency of pollutant photodegradation depends on the AC manufacturing process, the method of TiO_2_/AC synthesis, the operating conditions, and the target pollutants.

**Table 3:** Current publications on the uses of activated carbon in photocatalysis processes

Uses of activated carbon	Activated carbon type	Surface area	Reactive oxygen species (ROS)	Polluting substance studied	Operating conditions	Removal	Mineralization	Reference
Catalyst support	Powder activated carbon	156.2 m^2^/g	SO_4_^•–^ ^•^OH ^1^O_2_ h^+^	Sulfamerazine	Bi_2_O_3_-TiO_2_/AC dosage = 0.1 g/L Peroxydisulfate dose = 0.1 g/L pH = 7 Time = 120 min	97.96%	40%	Li et al. (2022)
	Powder activated carbon		^•^OH O_2_^•–^	Intracellular organic matter from algae extracted from Microcystis aeruginosa	Bi_2_O_3_-TiO_2_/AC dosage = 1.2 g/L pH = 7 Time = 20 min	DOC 64% UV_254_ 92% OD_680_ 100%		Wang et al. (2022)
Adsorbent	Bio-sourced activated carbon		^•^OH	Carbamazepine	TiO_2_- AC dosage = 0.13 g/L T = 10 °C pH = 9.5	70–80%		El mouchtari Em et al. (2022)

Jiang et al. (2023) compiled the following possible mechanisms of reactive oxygen species (ROS) formation in the photocatalytic process using AC as a catalyst, see Eqs. 1–8.

1 $$\mathrm{AC\ semiconductor\ photocatalysts}+\mathrm{hv }\to {\mathrm{e}}^{-} + {\mathrm{h}}^{+}$$

2 $${e}^{- }+ {O}_{2} \to {O}_{2}^{*}$$

3 $${O}_{2}^{*}+ {H}_{2}O \to {H}_{2 }{O}^{*}+ {OH}^{-}$$

4 $${H}_{2 }{O}^{*}+ {H}_{2}O \to {H}_{2}{O}_{2}+ *OH$$

5 $${H}_{2}{O}_{2} \to 2*OH$$

6 $${h}^{+}+organic\ pollutant \to {H}_{2}O+C{O}_{2}$$

7 $${h}^{+}+ {H}_{2}O \to *OH+ {H}^{+}$$

8 $${h}^{+}+ O{H}^{-} \to *OH$$

In photocatalytic processes coupled with AC, variables such as contaminant concentration, pH, catalyst dose, temperature, effects of water matrices, temperature, and AC use cycles, among others, are being studied.

No country stands out in the investigations into photocatalytic processes incorporating AC; however, six of the ten countries that published the most have generated at least one scientific publication. Regarding patents, the countries that stand out the most in the generation of technological developments in photocatalysis with AC are China (3) and Korea (2).

#### Fenton processes

The Fenton process is an AOP that has been widely applied in the treatment of wastewater of different origins. In Fenton oxidation, organic matter and dyes can be degraded by producing OH radicals using H_2_O_2_ and Fe^2+^ at a low pH. In addition to the formation of OH radicals, Fenton oxidation generates a reaction that allows the regeneration of Fe^2+^ ions, although it is very slow, which significantly reduces the oxidation efficiency due to the lower formation of hydroxyl radicals (Çalık and Çifçi 2022).

**Fig. 6:**
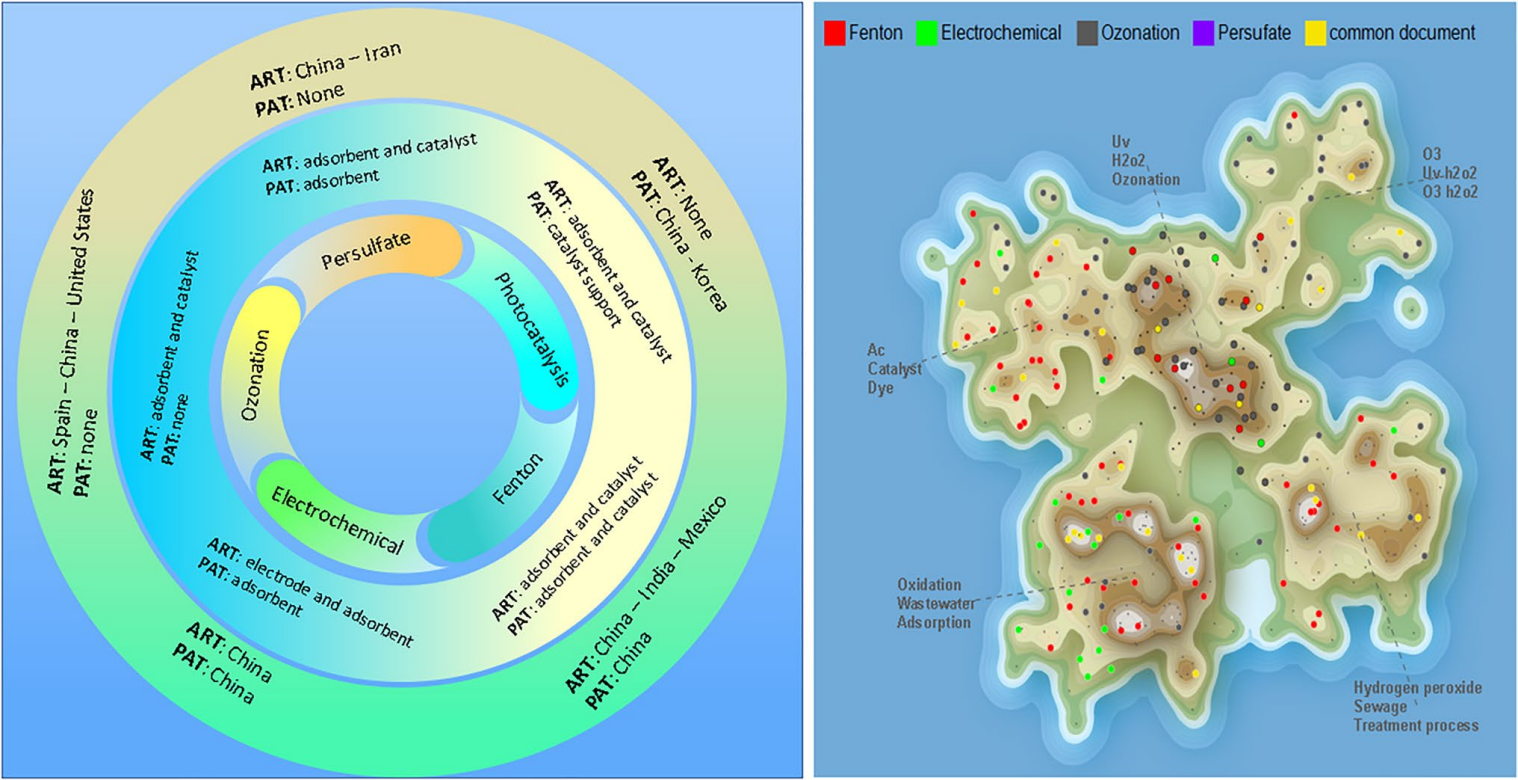
AC usage trends and AOPs distribution on the map

Research related to Fenton processes incorporating AC included 79 documents, mainly concentrated in two map areas (see Fig. 6). AC application in the Fenton processes is associated with the use of AC as an adsorbent (44%), as a catalyst (41%), as an electrode (10%), and as a catalyst support (5%). It was also found that patents represent 24% of the documents and scientific articles 76%. The above shows that Fenton-AC processes have been explored to treat different types of water and that applied knowledge has already been generated for technological development; of the AOPs reviewed in this study, this AOP has the highest number of patents (19 patents).

AC applications in Fenton processes have registered scientific publications and patents since 1995, although it has been since 2006 when documents are constantly generated. 2021 is the year with the highest number of documents (seven articles and five patents).

In scientific articles, the main applications of AC to Fenton processes are as a catalyst. Jiang et al. (2023) compiled the following possible mechanisms of ROS formation in the Fenton process using AC as a catalyst, see Eqs. 9–12.

9 $${Fe}^{2+}+ {H}_{2}{O}_{2} \to *OH+ {Fe}^{3+}$$

10 $${Fe}^{3+}+ {H}_{2}{O}_{2} \to {Fe}^{2+}+ {O}_{2}^{*-}$$

11 $$AC-OH+ {H}_{2}{O}_{2} \to COOH+ {H}_{2}O$$

12 $$AC-OH+ {H}_{2}{O}_{2} \to C{O}^{*}+ *OH+ {H}_{2}O$$

AC is also used as an adsorbent before and after the Fenton process, in which AC is used as an adsorbent for polluting substances, intermediate reaction products, or residual H_2_O_2_. AC is also used as a catalyst support (Fe, FeOx, Fe_3_O_4,_ Fe_3_O_4_-CeO_2_) and electrode in the electro-Fenton process. AC as an electrode has been investigated in Fenton processes as they provide a large specific surface area and high electrical conductivity when combined with iron nanoparticles or iron oxides (Dargahi et al. 2021). The use of AC as an electrode is also being investigated to improve the efficiency of the electro-Fenton process, in which AC electrodes are used to adsorb contaminants on the cathode, where ^•^OH is formed (Han et al. 2022).

**Fig. 7:**
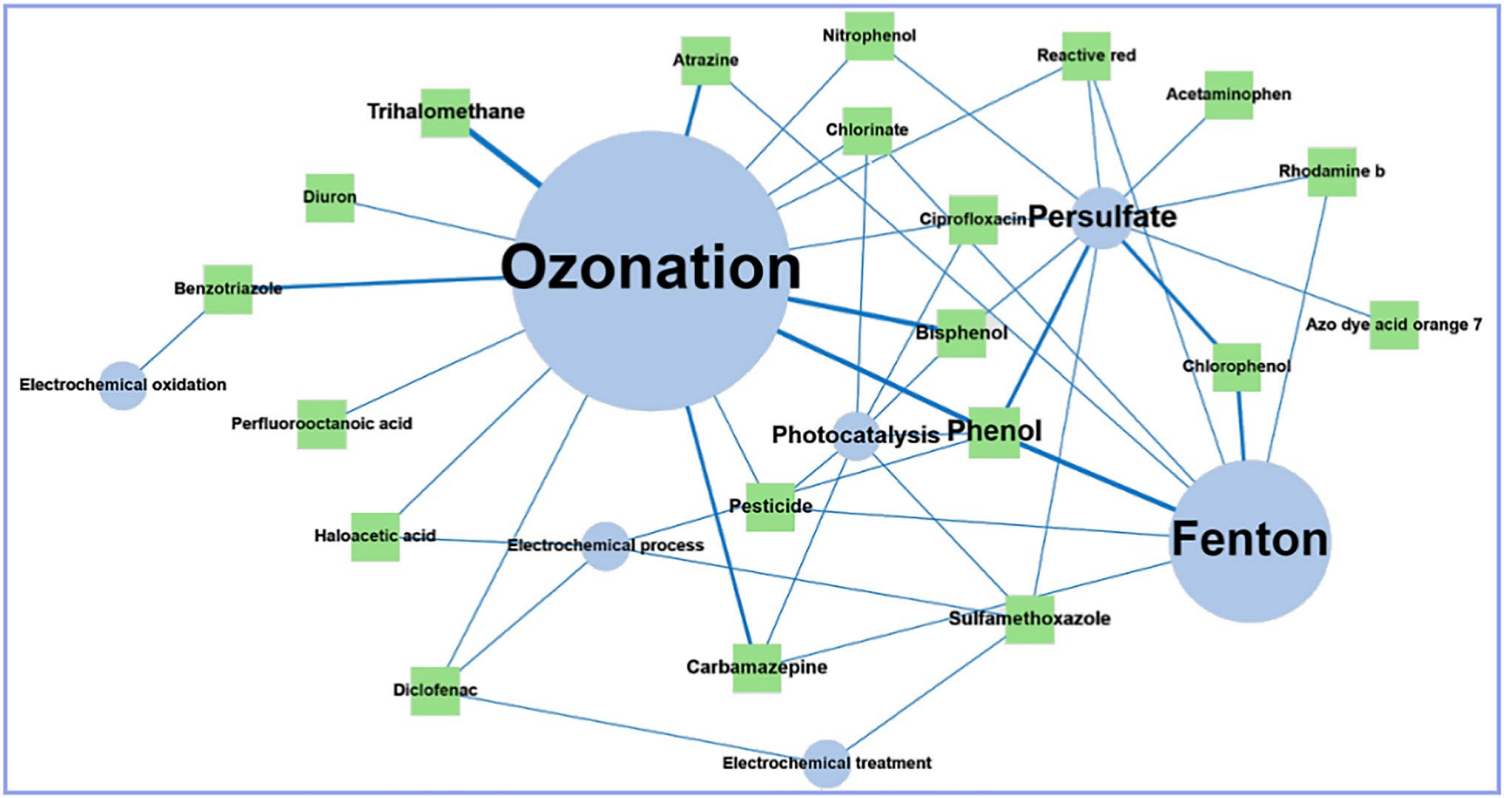
Main pollutants evaluated in scientific publications

Among the contaminants, most evaluated in the scientific investigations of the Fenton-AC processes are chlorophenol, phenol, rhodamine b, and reactive red (see Fig. 7).

The articles published in 2022 are detailed in Table 4, where it can be seen that there is a tendency to evaluate the degradation and/or mineralization of different dyes. In these recent investigations, AC is used as a catalyst, an adsorbent, and an electrode.

**Table 4:** Current publications on the uses of activated carbon in Fenton processes

Uses of activated carbon	Activated carbon type	Surface area	Reactive oxygen species (ROS)	Polluting substance studied	Operating conditions	Removal	Mineralization	Reference
Adsorbent/catalyst	Granular activated carbon	1602 m^2^/g		Aqueous solutions of the industrial dyes methylene blue, Eriochrome Black T and Ponceau S		95–99%		Gonçalves Gr et al. (2022)
Catalyst	Powder activated carbon	974 m^2^/g	^•^OH	Complex cork-boiling wastewater characterized by a high organic load and substantial content of phenolics	Liquid flow rate = 0.25 mL/min Mass of catalyst = 2.5 g [H_2_O_2_] = 9795 mg/L T = 100 °C pH = 3 Time = 72 h	COD = 78%	TOC = 58%	Garcia-Costa et al. (2022)
Adsorbent	Bio-sourced activated carbon			Dyes (Reactive Red 195, Synolon Brown S2, Orange Remazol RGB, Yellow Synozol K3, Reactive Orange, and Reactive Black 5)	AC dosage = 0.7 g/L [TiO_2_] = 20 mg/L [H_2_O_2_] = 1.2 mL/L	94.8–99.7%		Feuzer-matos et al. (2022)
Electrode/catalyst support			^•^OH	Reactive blue 19	[Reactive blue 19] = 100 mg/L Time = 60 min	⁓70%		Zhou et al. (2022)

Some of the variables evaluated in the Fenton processes coupled with AC were catalyst dosage, H_2_O_2_ concentration, pH, temperature, AC use cycles, liquid flow rate, among others. In general, the incorporation of AC in Fenton processes generates interesting results. For example, Feuzer-matos et al. (2022) compared the dye removal efficiency with some AOPs; the best efficiency was obtained by incorporating AC. Garcia-Costa et al. (2022) compared different catalysts in wastewater treatment; Fe showed a significant catalytic activity, but high Fe leaching occurred. In contrast, AC is a stable option showing interesting removal efficiencies (COD 78%, TOC 58%). Zhou et al. (2022) compared the efficiency of a conventional Pd cathode with a hybrid Pd/AC cathode. This study found that the Pd/AC cathode obtained a H_2_O_2_ concentration 248.2% higher than the Pd cathode and the O_2_ utilization efficiency increased from 3.2 to 10.8%.

The ten countries that generate the most publications have studied Fenton processes in scientific publications. The countries that most investigated Fenton processes with AC are China (13.9%), India (11.4%), and Mexico (6.3%); however, there is no evidence of a university that stands out in publications on this topic. On the other hand, this AOP is the most relevant for India with respect to the other processes analyzed (ozonation, electrochemical, persulfate, and photocatalysis).

#### Electrochemical processes

Electrochemistry involves chemical phenomena related to charge differences in a liquid medium or solution (Bekmezci et al. 2021). Most electrochemical processes are governed by the transfer of electrons between redox species. The interface where electron transfer occurs is crucial to regulate and optimize the electrochemical reaction system (Long and Tian 2020). In general, the degradation mechanism with electrochemical processes is described in Eqs. 13–16 (Lian et al. 2020).

13 $${O}_{2}+ {2H}^{+}+ {2e}^{-} \to {H}_{2}{O}_{2}$$

14 $${{H}_{2}O}_{2}+ {Fe}^{2+} \to *OH+ {Fe}^{3+}+ {OH}^{-}$$

15 $${Fe}^{3+}+ {e}^{-} \to {Fe}^{2+}$$

In the current search, 40 documents were obtained that related electrochemistry with AC, which are distributed in 4 regions of the map, but 50% of the documents are concentrated in a single region (see Fig. 6). The main applications of AC in electrochemical processes are as an electrode (46%) and an adsorbent (38%).

Of the 40 documents found, patents represent 20% of the documents and scientific articles 80%. AC applications in electrochemical processes have registered scientific publications and patents since 2006, with 2020 being the year with the largest number of documents (five articles and one patent).

According to scientific articles, AC is incorporated into electrochemical processes as an electrode, an adsorbent, and a catalyst. However, no trend exists toward evaluating a particular pollutant (see Fig. 7).

The applications of AC in electrochemical processes in articles published in 2022 are detailed in Table 5.

**Table 5:** Current publications on the uses of activated carbon in electrochemical processes

Uses of activated carbon	Activated carbon type	Surface area	Reactive oxygen species (ROS)	Polluting substance studied	Operating conditions	Removal	Mineralization	Reference
Electrode/adsorbent	Biochar	62 m^2^/g	^•^OH	Diclofenac and sulfamethoxazole	Current density = 7 mA/cm^2^ Biochar dosage = 30 mg [Diclofenac] = 10 mg/L [Sulfamethoxazol] = 10 mg/L Inter-electrode distance = 7.5 cm pH = 7 Time = 30 min	Runs in wastewater effluents 49%—diclofenac 86%—sulfamethoxazole		Soares et al. (2022)

In scientific publications, electrochemical processes have been studied by six of the ten countries that published the most in this field. China stands out the most in research on electrochemical processes with AC (12.5%).

#### Ozonation

Ozonation is an efficient AOP for degrading organic pollutants in effluents since ozone and hydroxyl radicals have a high oxidizing power (2.07 V and 2.8 V). Studies have shown that including a catalyst (catalytic ozonation) improves the degree of mineralization because the catalyst allows the formation of highly reactive radicals (Hernandez et al. 2019). Ozonation has been applied for the treatment of drinking water for disinfection and oxidation, and it is also used for wastewater treatment and drinking water reuse (Lim et al. 2022).

A total of 88 documents were found that related ozonation with AC. These documents are located in five map regions but are mainly concentrated in two areas (see Fig. 6). Some documents extend to other regions of the map, which could mean that other topics are related to the application of AC to ozonation processes.

Patents only represent 3.4% of all documents; this could happen because the knowledge generated in the investigations is still in a growth stage and a considerable advantage has not yet been developed to take them to a large scale. The ozonation processes with AC are one of the AOPs that generate the most scientific publications, which mean that the scientific community is interested in this topic. This is the AOP-AC that most explores the degradation and removal of emerging contaminants; among the emerging contaminants evaluated in scientific publications are carbamazepine, diclofenac, ciprofloxacin, naproxen, trimethoprim, bisphenol, benzotriazole, chlorothalonil, hydrochlorothiazide, tramadol, levetiracetam, sotalol, irbesartan, ethinylestradiol, atrazine, diuron, among others (see Fig. 7). This interest could lead to more patents in a few years.

AC applications in ozonation processes have registered scientific publications and patents since 1991, although it has been since 2008 when documents were constantly generated. The years 2020 and 2021 are the ones that register the largest number of documents (nine articles each year).

Researchers have used AC in ozonation processes in different ways to reduce operating costs and increase process efficiency. According to the articles found, AC is used as an adsorbent in processes before and after the ozonation process (48%). In other studies, AC has been used as a catalyst (48%) in the ozonation process.

AC as an adsorbent has shown promising results in systems coupled with ozone, where AC adsorbs the byproducts generated by the ozonation process. For drinking water treatment, for example, excellent reduction of trihalomethane (THMFP) and haloacetic acid (HAAFP) has been evidenced (Phan et al. 2023). On the other hand, catalytic ozonation using AC as a catalyst is a technique that not only provides the advantage of high adsorption capacity of AC for pollutant removal, but also serves as an initiator/promoter in an O_3_ decomposition radical-type chain reaction that enhances the formation of ^•^OH (Vatankhah et al. 2019). Functional groups on the AC surface are relevant for specific catalytic reactions. The type and concentration of such functional groups determine the application of catalysts (Matos et al. 2017). Some authors indicate that the presence of nitrogen and oxygen functional groups in AC and the high surface area are the main characteristics of AC to initiate the decomposition of O_3_ into ^•^OH. In turn, the nitrogen functional groups contribute to increase the electron density, accelerating the decomposition of O_3_ to ^•^OH. In contrast, the oxygen functional groups initialize a chain reaction mechanism and provide adsorption sites for surface reactions (Vatankhah et al. 2019). Alameddine et al. (2021) propose the following mechanism for catalytic ozonation using AC as a catalyst, see Eqs. 16–21.

16 $${O}_{3}+ {H}_{2}O \to *OH+ {O}_{2}$$

17 $${O}_{3}+ {OH}^{-} \to {O}_{2}^{*-}+ {HO}_{2}^{*}+ {HO}_{3}^{*}+ {O}_{3}^{*-}$$

18 $${2O}_{2}^{*-}+ {2H}^{+} \to {1}^{1}{O}_{2}+ {H}_{2}{O}_{2}$$

19 $${O}_{3}+ {HO}_{2}^{*} \to *OH+ 2{O}_{2}$$

20 $$AC+ {H}_{2}O \to AC-OH+ {H}^{+}$$

21 $${O}_{3}+ AC-OH \to *OH; {1}^{1}{O}_{2}; {O}_{2}^{*-}$$

Table 6 shows AC applications in ozonization processes published in 2022, where the use of AC as a catalyst and an adsorbent stands out.

**Table 6:** Current publications on the uses of activated carbon in ozonation processes

Uses of activated carbon	Activated carbon type	Surface area	Reactive oxygen species (ROS)	Polluting substance studied	Operating conditions	Removal	Mineralization	Reference
Adsorbent	Granular activated carbon	1000 m^2^/g	^•^OH	Recalcitrant contaminants from the leachate	[O_3_] = 1000 mg/L AC density = 1 g/mL Time = 33.77 min	COD = 55.2% TDS = 54.4% BOD = 56.5%		Nabavi et al. (2022)
Catalyst	Powder activated carbon	710 m^2^/g	^•^OH	Neonicotinoids	Initial concentration of each compound = 1 μM T = 20 °C pH = 7 Time = 5 min	Several water matrices 20–63%		Real Fj et al. (2022)
Catalyst support	Powder activated carbon	32.4 m^2^/g	^•^OH	Bisphenol A	AC/CeO_2_/ZnO dosage = 500 μg/L pH = 8 Time = 60 min	97%	TOC = 61%	Pokkiladathu et al. (2022a)
	Powder activated carbon		^•^OH	Bisphenol A	AC/Cu_2_O/ZnO dosage = 650 µg/L pH = 8 Time = 60 min	98%		Pokkiladathu et al. (2022b)
	Powder activated carbon		^•^OH	Bisphenol A	AC/Bi_2_O_3_/V_2_O_5_ dosage = 500 µg/L [Bisphenol A] = 5 mg/L pH = 8 Time = 60 min	97%	TOC = 68%	Pokkiladathu et al. (2022c)
Adsorbent/catalyst	Granular activated carbon	1473 m^2^/g	^•^OH	Penicillin G	O_3_ flow rate = 0.8 mg/min [Penicillin G] = 50 mg/L AC dosage = 50 mg/L pH = 10 Time = 40 min	100%	TOC = 52%	Hekmatshoar et al. (2022)
	Granular activated carbon	1169.84 m^2^/g		Alkylphenols and their ethoxylates		87.7%—4-nonylphenols 100%—other alkylphenol derivatives	TOC = 65.7%	Cižmárová et al. (2022)

In ozonation processes coupled with AC, variables such as ozone concentration, pollutant concentration, catalyst dosage, and pH, among others, are being studied. Incorporating AC in ozonation processes presents interesting results of pollutant removal, allowing the continuity of research in this area. For example, Cižmárová et al. (2022) performed catalytic ozonation studies with Fe-modified zeolite, Mn-modified zeolite, and AC; the highest removal efficiencies were with AC, 87.7% for 4-nonylphenols, 100% for other alkylphenol derivatives. On the other hand, Nabavi et al. (2022) obtained better results incorporating AC as an adsorbent to the ozonation process (COD 55.2%, TDS 54.4%, and BOD 56.5%) than the ozonation process alone (COD 25.1%, TDS 25.7%, and BOD 43.6%). Studies by Pokkiladathu H et al. (2022c) show that catalytic ozonation with AC/Bi_2_O_3_/V_2_O_5_ catalyst is 32% more efficient in TOC analysis than non-catalytic ozonation. Hekmatshoar et al. (2022) evaluated adsorption processes with AC, ozonation processes and catalytic ozonation processes with AC for penicillin G; the removal efficiencies were 11.7%, 33.6%, and 85%, respectively.

In scientific publications, ozonation is a process studied by the ten countries that generate the most publications. The countries that most investigate the ozonation process incorporating AC are Spain (15.9%), China (14.8%), and the USA (10.2%); the universities that stand out the most in the development of research in this process are The University of Extremadura-Badajoz and the University of Granada in Spain, University of Beijing in China, and the University of California in the United States.

#### Persulfate

Persulfate-based oxidation is an AOP with high oxidizing power (Eo = 2.12 V), which can produce ROS, such as sulfate radical (SO_4_^•–^), hydroxyl radical (^•^OH), or superoxide radical (O_2_^•–^), and non-radical species known as singlet oxygen (^1^O_2_). Thermal methods, UV radiation, metal-loaded carbon-based material, or transition metals can activate persulfate (Annamalai et al. 2022).

AC applications in persulfate processes have registered scientific publications and patents since 2008, although it has been since 2017 when more interest began to be generated and documents were constantly published. The largest number of articles published (nine) is recorded in 2022.

In the search process, 35 documents related to persulfate-based AOPs with the incorporation of AC were found, which are located mainly in two regions of the map (see Fig. 6). According to the scientific articles found, AC has been investigated primarily to activate persulfate through the carbocatalysis reaction, where AC is used as a catalyst. In persulfate system–based AOPs—AC, pollutants can be degraded via the radical and non-radical pathways. Through the radical path, SO_4_^•–^ and ^•^OH are produced by reacting the active sites of the AC with persulfate (Honarmandrad et al. 2023). The active sites of AC that contribute to the activation of persulfate include surface functional groups, delocalized π electrons, sp^2^ hybrid carbon at defect edges, and oxygen-containing functional groups (–COOH, C–OH, C = O) (Gao et al. 2022). On the other hand, the characteristics of AC, such as the large specific surface area and the porous structure, play a crucial role in the non-radial mechanism, promoting a direct transfer of electrons from the pollutants to the oxidant (Annamalai et al. 2022). Jiang et al. (2023) compiled the following proposals for persulfate-based AOP mechanisms using AC as a catalyst, see Eqs. 22–33.

22 $$AC{-}_{surface}-OH+ {S}_{2}{O}_{8}^{2-} \to AC {-}_{surface}-{O}^{*}+ {SO}_{4}^{*-}+ {HSO}_{4}^{-}$$

23 $$AC{-}_{surface}-OOH+ {S}_{2}{O}_{8}^{2-} \to AC {-}_{surface}-{OO}^{*}+ {SO}_{4}^{*-}+ {HSO}_{4}^{-}$$

24 $${HSO}_{5}^{-}+AC{-}_{defect} \to {OH}^{-}+ {SO}_{4}^{*-}+ {AC}_{defect}^{+}$$

25 $${HSO}_{5}^{-}+AC{-}_{defect} \to *OH+ {SO}_{4}^{2-}+ {AC}_{defect}^{+}$$

26 $$Persistent\ free\ radicals+{e}^{-}+{O}_{2} \to AC- Persistent\ free\ radicals+ {O}_{2}^{*-}$$

27 $${HSO}_{5}^{-}+ {O}_{2}^{*-} \to {SO}_{4}^{*-}+ {O}_{2}+ *OH$$

28 $${O}_{2}^{*-}+ {H}^{+} \to {1}^{1}{O}_{2}+ {H}_{2}{O}_{2}$$

29 $${2O}_{2}^{*-}+ {2H}_{2}O \to {1}^{1}{O}_{2}+ {2OH}^{-} {+H}_{2}{O}_{2}$$

30 $${O}_{2}^{*-}+ {SO}_{4}^{*-} \to {1}^{1}{O}_{2}+ {SO}_{4}^{2-}$$

31 $${O}_{2}^{*-}+ {O}_{2}^{*} \to {1}^{1}{O}_{2}+ {H}_{2}{O}_{2}$$

32 $${HSO}_{5}^{-} \to {SO}_{5}^{*-}+{H}^{+}+ {e}^{-}$$

33 $${SO}_{5}^{*-}+ {H}_{2}O \to {1}^{1}{O}_{2}+ {HSO}_{4}^{-}$$

Among the contaminants most evaluated in scientific research related to persulfate-AC processes are phenol, bisphenol, nitrophenol, chlorophenol, and azo dye (see Fig. 6). The articles published in 2022 are shown in Table 7, where AC is used as a catalyst, an adsorbent, and a catalyst support.

**Table 7:** Current publications on the uses of activated carbon in persulfate-based oxidation

Uses of activated carbon	Activated carbon type	Surface area	Reactive oxygen species (ROS)	Polluting substance studied	Operating conditions	Removal	Mineralization	Reference
Catalyst			SO_4_^•–^ ^•^OH	Acetaminophen	AC dosage = 8.25 mM [Persulfate] = 1.65 mM pH = 7 Time = 90 min	100%	TOC = 24%	Qutob et al. (2022)
	Powder activated carbon	950 m^2^/g	SO_4_^•–^ ^•^OH	Sulfamethoxazole	AC dosage = 100 mg/L [Persulfate] = 5 mM pH = 4		TOC = 98.5%	Liang et al. (2022)
	Activated carbon fiber		SO_4_^•–^ ^•^OH	Bio-treated coking wastewater	[Peroxymonosulfate] = 8 mM T = 30 °C pH = 7 Time = 120 min	COD = 88.7%		Su et al. (2022)
	Powder activated carbon	1086 m^2^/g	SO_4_^•–^ ^•^OH ^1^O_2_	Phenol	AC dosage = 2.5 mg/g [Persulfate] = 5 mM Solid–liquid ratio = 1:5 T = 25 °C pH = 3–11 Time = 24 h		TOC = 92.4%	Annamalai et al. (2022)
Catalyst support	Granular activated carbon	482.61 m^2^/g	SO_4_^•–^ ^•^OH	P-nitrophenol	Co-nZVI/AC dosage = 1.5 g/L [P-nitrophenol] = 15 mg/L [Persulfate] = 1 mM pH = 6 Time = 6 h	99.19%		Zhang et al. (2022)
	Powder activated carbon	1307 m^2^/g	^1^O_2_ SO_4_^•–^ ^•^OH	Rhodamine B	FeC_2_O_4_/AC dosage = 0.2 g/L [Rhodamine B] = 300 mg/L [Peroxymonosulfate] = 500 mg/L T = 25 °C pH = 4 Time = 150 min	97%		Xue et al. (2022)
		515.46 m^2^/g	SO_4_^•–^ ^•^OH	Ciprofloxacin	Co-AC dosage = 0.75 g/L [Persulfate] = 2 mM [Ciprofloxacin] = 50 μM T = 25 °C pH = 6 Time = 120 min	100%		Erdem and Erdem (2022)
Adsorbent/catalyst	Powder activated carbon	1363.4 m^2^/g	SO_4_^•–^ ^•^OH	4-chlorophenol	Magnetized AC dosage = 1250 mg/L [Persulfate] = 350 mg/L [4-chlorophenol] = 100 mg/L pH = 5 Time = 60 min	99.5%		Hadi et al. (2022)

In persulfate-based AOPs coupled with AC, variables such as persulfate concentration, contaminant concentration, catalyst dosage, pH, catalyst use cycles, coexisting ions, and temperature, among others, are being studied. Systems involving persulfate-based AOP and AC show better efficiency than working the processes separately. For example, Zhang et al. (2022) showed that the Co-nZVI catalyst supported on AC in the presence of persulfate achieved a degradation of 99.19%, while the catalyst alone degraded 69.8%. Erdem and Erdem (2022) also showed a synergistic effect between the Co-AC catalyst on persulfate-based AOPs, obtaining 100% degradation; with only persulfate, the degradation reached 40%.

In scientific publications, persulfate-based processes are studied by eight of the top ten countries that generate the most publications. China (42.8%) and Iran (17.1%) are the main countries investigating persulfate-AC systems. The universities that stand out the most are the University of Beijing and the University of Shandong.

## Technological maturity

**Fig. 8:**
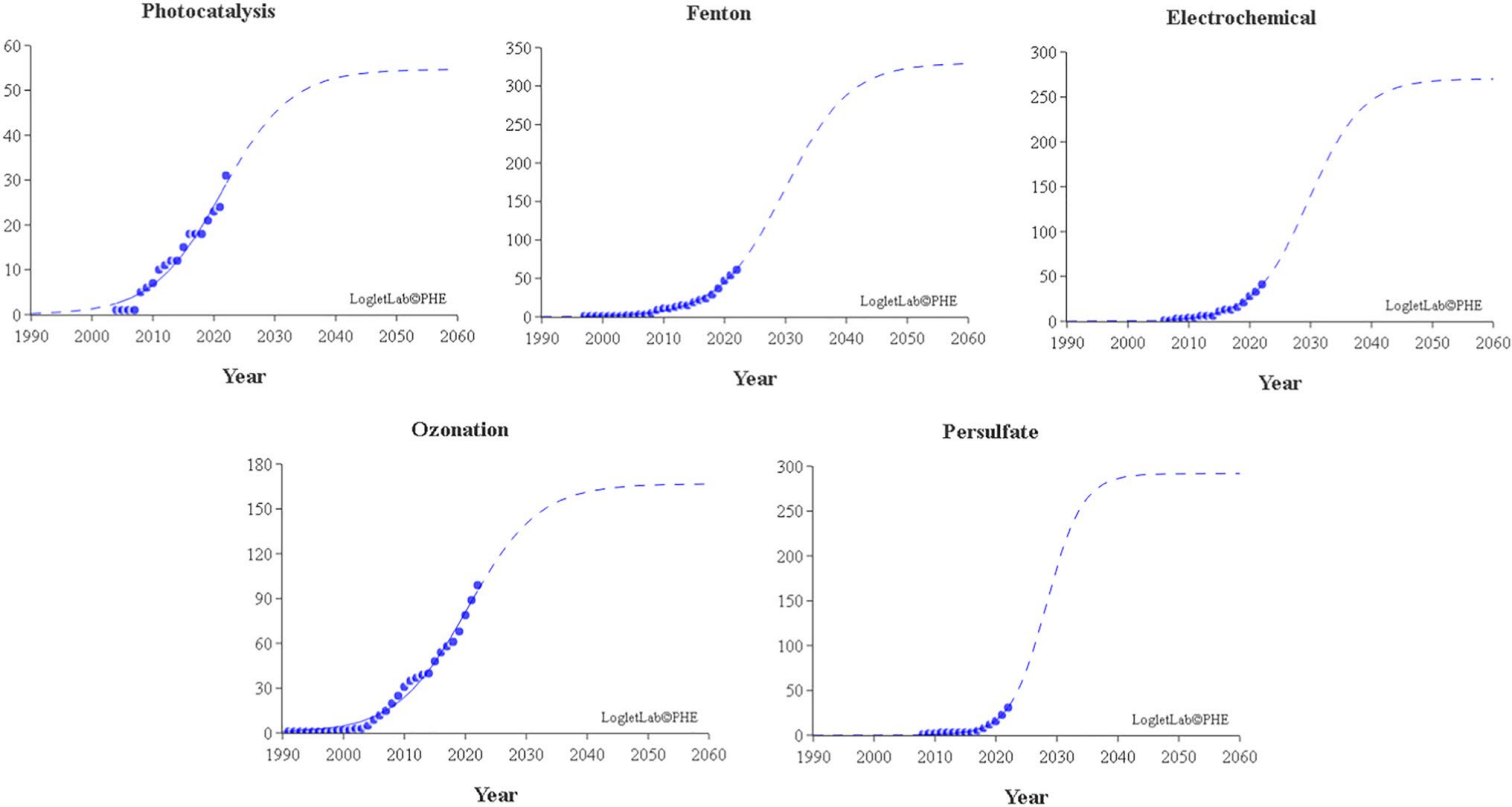
Technological maturity of AOPs involving AC: photocatalysis, Fenton, electrochemistry, ozonation, and persulfate

Through Loglet Lab4 software, a projection of future scientific publications was carried out to identify the degree of technological maturity. The following AOPs involving AC were analyzed: photocatalysis, Fenton, electrochemistry, ozonation, and persulfate. The input data were the cumulative number of publications related to each AOP from 1991 to 2022. Subsequently, the data were fit to a logistic regression using the least squares method (see Fig. 8).

Photocatalysis, Fenton, electrochemical, ozonation, and persulfate technologies are in a growth stage and will reach maturity in 2034, 2042, 2040, 2034, and 2035, respectively. It is expected that more patents will be generated when these technologies’ scientific publications reach maturity. According to Fig. 8, the persulfate-based AOP is the process that has had the most significant growth in scientific publications in recent years (90% of the publications have been generated from 2017 to 2022); this shows a tremendous current interest by the scientific community in the research of this AOP. For this reason, it is one of the AOPs that will reach maturity more quickly in scientific publications, despite being one of the AOPs that began to be published more recently. This high interest in persulfate-based AOPs with AC may be related to the advantages that this AOP has since it can degrade pollutants via the radical pathway (^•^OH, SO_4_^•–^) and by the non-radical pathway (electron transfer, singlet oxygen, and direct oxidation). SO_4_^•–^ is a strong oxidant with a higher standard redox potential than ^•^OH; it also has a long half-life 30 times higher than ^•^OH, allowing more contact with contaminants. On the other hand, the non-radical pathway allows working in a wider pH range than the radical pathway; the oxidation capacity of persulfate could be fully developed in the non-radical pathway and the self-quenching of the generated radicals could also be avoided (Gao et al. 2022).

AC applications that stand out in the scientific investigation of photocatalysis are as an adsorbent and a catalyst. However, the dynamics of article publication is slow. Possibly, future research is oriented to evaluate photocatalytic-AC systems coupled with other AOPs (as seen in the “Photocatalysis” section).

AC applications that stand out in the scientific investigation of the Fenton processes are as an adsorbent and a catalyst. The applications of AC that stand out in the patents are as an adsorbent, a catalyst, and an electrode. As new knowledge develops, more patents are expected to be generated in applications as adsorbents and catalysts in Fenton processes.

AC applications that stand out in the scientific investigation of electrochemical processes are as an adsorbent and an electrode. In contrast, the application of AC that stands out in the patents is an adsorbent. It is expected to increase the number of patents in electrochemical processes with the applications of AC as an adsorbent and to generate other patents where AC is used as an electrode.

AC applications that stand out in scientific research on ozonation processes are as an adsorbent and a catalyst, while no application stands out for patents. Among the AOPs analyzed in this study, ozonation is the process that has generated the fewest patents. It is expected that the knowledge generated in scientific research may generate in the future a greater number of patents in the applications of AC as an adsorbent and as a catalyst.

AC applications that stand out in the scientific research of persulfate-based AOPs are as a catalyst and an adsorbent, while the application of AC that stands out in the patents is as an adsorbent. In the future, it is expected to generate more patents in the applications of AC as a catalyst and an adsorbent.

## Conclusion

AOPs incorporating AC in water treatment systems have been developed since 1991, with China being the leading country in scientific publications and patents. Although the first article and first patent were reported in 2004, consistent production of scientific articles started in 2009. The connection between Chinese universities has made it strong in this area, making it possible to strengthen and patent knowledge. The development of joint research may be a strategy that has been influenced by government policies aimed at water protection that have been enacted in the last two decades.

Of the AOP-ACs analyzed in this article, ozonation and persulfate-based AOP have generated the most interest in the scientific community in recent years (from 2017 to 2022). For this period, persulfate-based AOP has the highest growth in scientific publications. According to Fig. 8—technological maturity, research on persulfate-based AOPs is expected to increase faster than other AOPs (it has a steeper slope). This high interest in persulfate-based AOP could be related to the synergy that this process has with CA to generate ROS and degrade pollutants via radical and non-radical.

For the Fenton process, ozonation, and persulfate-based AOP, the main uses of AC in scientific research are as an adsorbent and a catalyst, while for the electrochemical process, the main uses are as an electrode and an adsorbent. However, the Fenton, ozonation, and persulfate processes have also generated research on the use of AC as an electrode, but in smaller proportions, which indicates that electro-Fenton processes, electrochemical ozonation, and electro oxidation are being explored.

The use of AC as an adsorbent is the most common application in the AOPs analyzed in this study; this may be because the properties of AC as an adsorbent have been studied for a long time, and this knowledge is used to improve the efficiency of the process and to be able to scale it to a larger size. All the AOPs analyzed in this study have generated scientific publications related to AC as an adsorbent (121 articles), where the ozonation process is the one that has been most investigated. In the case of patents, the use of AC as an adsorbent for the AOPs analyzed in this article also stands out, but the ozonation process is the only one that has not generated patents.

In this sense, scientific collaboration can be one of the ways to achieve the technology development. In the AOPs-AC reviewed in this article, some collaborative networks are highlighted, where the main interest is the research of ozonation and persulfate processes. The main universities that lead these collaborative networks are the University of Beijing (ozonation y persulfate), the University of Extremadura-Badajoz (ozonation), the University of California (ozonation), the National Center for Scientific Research (ozonation), University of Granada (ozonation), and University of Shandong (persulfate). It is expected that in the future, patents will be generated in the ozonation and persulfate process where AC is used as an adsorbent and/or catalyst.

The AOP-AC that is being most investigated for the reduction and/or degradation of different pollutants is ozonation; this could be related to the versatility, efficiency, and/or flexibility of this system to treat different types of pollutants; however, the ozonation-AC process has not yet generated patents. Possibly, researchers are reviewing the technical and economic feasibility of this technology on a large scale in order to commercialize it.

One of the most studied process conditions in AOPs is the catalyst dose, given that a higher dose favors the generation of ROS; however, an excessive dose of AC (as a catalyst) leads to self-quenching free radicals. In photocatalytic processes, they can also generate lower performance because it prevents the entry of light. Another trend is research on the number of cycles that the AC can have with high performance; this leads to research aimed at a larger scale with a focus on sustainability and lower implementation costs.

## Data Availability

No data was used for the research described in the article.
